# Understanding the Thickness Effect on the Tensile Strength Property of Dyneema^®^HB26 Laminates

**DOI:** 10.3390/ma11081431

**Published:** 2018-08-14

**Authors:** Lorenzo Iannucci, Stefano Del Rosso, Paul T. Curtis, Dan J. Pope, Phillip W. Duke

**Affiliations:** 1 Department of Aeronautics, Imperial College London, London SW7 2AZ, UK; stefano.delrosso@imperial.ac.uk (S.D.R.); p.curtis@imperial.ac.uk (P.T.C); 2 Defence Science and Technology Laboratory, Porton Down, Salisbury SP4 0JQ, UK; DJPOPE@mail.dstl.gov.uk (D.J.P.); PWDUKE@mail.dstl.gov.uk (P.W.D.)

**Keywords:** finite element (FE), mechanical tests, ultra-high molecular weight polyethylene

## Abstract

In this study, an experimental and numerical investigation is presented on the effect of thickness and test rate within the pseudo static regime on the tensile properties of Dyneema^®^HB26 laminates. A detailed experimental presentation on the tensile testing of different thickness is presented and highlights the commonly seen observation that the tensile strength of a laminate reduces as a function of the specimen thickness. To understand these experimental observations, a constitutive material model of the individual macro fibril is developed and applied to modelling the fibre and upscaling to the laminate. The modelling strategy is implemented into ls-dyna and used to perform a parameter study on the specimen geometries used in the experimental study. The model assumes that the fibril strength is a function of the amorphous volume within the fibre and hence fibril. It can be observed that the experimental behaviour can be simulated by modelling the interface between laminate plies and the fibril, and hence fibre failure. The weak interfaces from the fibril to the laminate scale make the testing of fibres and laminates very difficult. Hence, it is proposed that the intrinsic fibril strength should be used as a measure of strength, and the fundamental strength is determined through numerical studies.

## 1. Introduction

**Figure 1:**
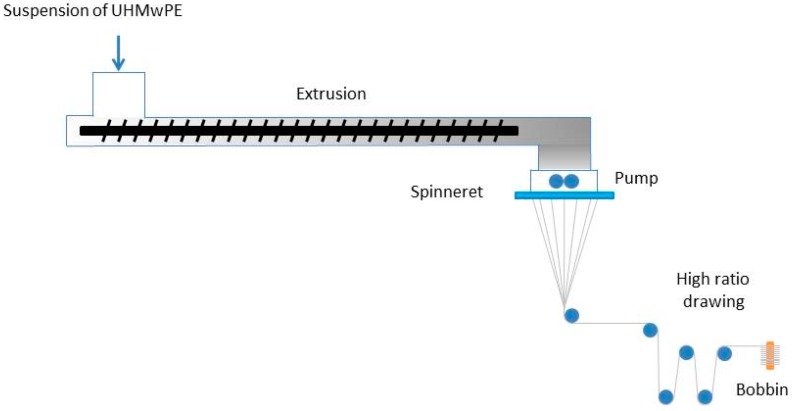
Gel spinning process.

High performance fibres made of gel-spun ultra-high molecular weight polyethylene (UHMwPE) have been increasingly used in multiple applications, such as ballistic protection, since their first synthesis in the 1980s. DSM commercialised UHMwPE fibres under the trade name Dyneema^®^ in the late 1970s [1]. Dyneema^®^ fibres are manufactured via the gel-spinning process (Figure 1). A solution of polyethylene having very long polymeric chains is continuously extruded and the chains are partially aligned when forced through the spinneret. The solution is then cooled down, the material starts to crystallise, and the solvent is removed. During the drawing stage, the molecules are further stretched and aligned to the fibre axis. Thus, the filaments are gathered together and wound over a cylindrical support. Over the years, modifications and improvements of the manufacturing process have been applied to the production of different grades of yarns, each with a unique combination of properties [2,3]. The molecules within these fibres have a typical length of 36 μm [4].

Dyneema^®^ fibres are used in many different applications from cut-resistant gloves, ropes, and nets to high performance textiles for sailing, from bio-compatible medical devices, such as stents, ligament replacement, and sutures [5], to soft and hard armours [6,7,8]. Dyneema^®^HB26 is a hard ballistic grade of composite made of Dyneema^®^SK76 fibres embedded in a polyurethane resin system, arranged in a [0°/90°]_2_ laminated prepreg with a fibre volume fraction $V_{f\;}\sim \;83\%$ [9]. The prepregs can be stacked and consolidated to create laminates of desired thickness.

**Figure 2:**
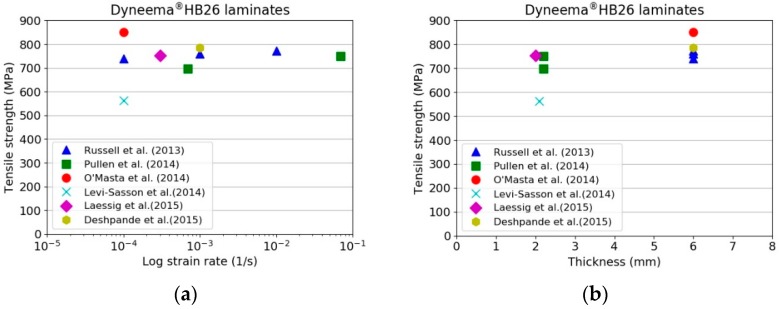

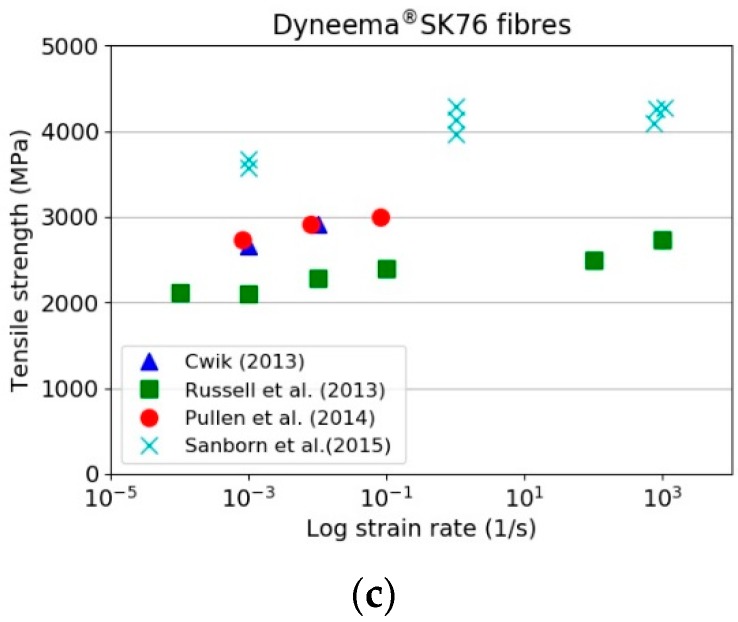
Tensile strength of Dyneema^®^HB26 laminates as function of: (**a**) Strain rate; (**b**) Thickness; (**c**) Tensile properties of Dyneema^®^SK76 fibres.

The tensile properties of Dyneema^®^SK76 fibres are commonly available in the open literature. However, test results sometimes differ from different sources, and are often a function of the specimen sizes. This is generally associated with different testing methods and loading/support conditions employed. Nevertheless, it is generally accepted that the fibres are viscoelastic and their mechanical properties dependent on the testing conditions. The higher the strain rate, the lower the temperature, the higher the tensile strength and the lower the strain to failure. On the other hand, the lower the strain rate, the higher the temperature, the lower the strength and the higher the strain to failure. Figure 2 shows the tensile properties of Dyneema^®^SK76 fibres and its laminates gathered from the literature [9,10,11,12,13,14,15,16]. It should also be noted that the in-plane and out-of-plane shear strengths are very low, thus the transfer of loads between the different scales requires a much longer length than conventional materials; this load transfer between fibre and resin within a composite is sometimes referred to as a shear-lag.

Russell et al. [9] noted that the failure strength and strain to failure of the yarn is 20% and 30–50% greater than the failure strength and strain to failure of the laminate, respectively. They attributed these differences to the changes in the morphology of the fibres during the consolidation process. O’Masta et al. [11] attributed the reduction in strength to the fibre waviness developed during the manufacture of the prepregs and non-uniform loading of the fibres within the composite. From tests performed on specimens cut from HB26 laminates, they calculated an ultimate tensile strength of $\sigma_{uts}=$ 850 MPa. Levi-Lasson et al. [12] performed tensile tests on thick specimens from HB26 laminates having different geometries. The authors demonstrated the difficulty of performing successful tensile tests on specimens having rectangular and standard dog-bone geometries. In both the investigated cases, separation of the outer layers in contact with the grips from the internal ones was observed. They developed a new specimen geometry, like a bow-tie with a set of 10 bolts, in order to assist the load transfer from the gripped area to the gauge region. Nevertheless, the maximum tensile strength was noted to be around 560 MPa. They attributed this to the low ability of load transfer between the fibres and matrix in the composite. Lässig et al. [13] performed tensile tests on 2 mm thick HB26 laminates using the specimen geometry adopted by Russell et al. [9], noting a tensile strength of the laminate as low as 31% with respect to the reference test [9]. As discussed in [9,12], it is very difficult to introduce axial stresses from the tabbed regions of the specimen into all layers and fibres within the gauge length by shear transfer, especially for a material which exhibits a very low and non-linear shear strength between layers (out of plane) and between fibres (in-plane). Such materials also exhibit low frictional behaviour, hence the load transfer between layers and fibres (debonding) is poor as well.

**Figure 3:**
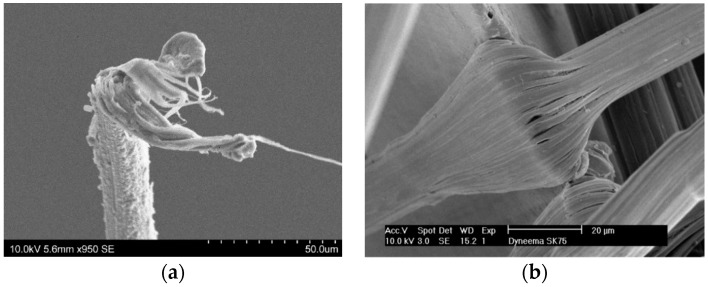
(**a**) Failure of an SK76 filament (note individual softening and drawing of fibrils); (**b**) SK76 filament over a razor blade (reproduced from Reference [18]). Note debonding of individual fibrils.

To understand the tensile properties of Dyneema^®^ fibres, plies, and laminates, it is proposed that the only relevant parameter of interest is the macro fibril strength. It can also be argued that during a ballistic impact the projectile only experiences the macro fibril strength, not the laboratory measured fibre, ply, or laminate strengths, which are always much lower than the macro fibril strength. It is also proposed that failure of the fibre will always occur at the macro fibril scale via internal friction within the amorphous regions due to chain slippage, leading to a highly localised adiabatic heating and softening, followed by localised failure of the material. This is illustrated in Figure 3a, which shows the drawing of the macro fibrils during failure. Detailed fractography failure surfaces of Dyneema^®^ are shown in Greenhalgh et al. [17] for a series of ballistic impacts. Conclusions from [17] indicate that the macrofibril failure, similar to Figure 3a, occurs away from the impact site at the support locations. The weak macro fibrils can be seen in Figure 3b [18] and clearly shows the macro fibrils debond readily. A new constitutive material model for the macro fibril is developed based on these observations and is extended to the fibre and laminate.

## 2. Materials and Experimental Methods

**Figure 4:**
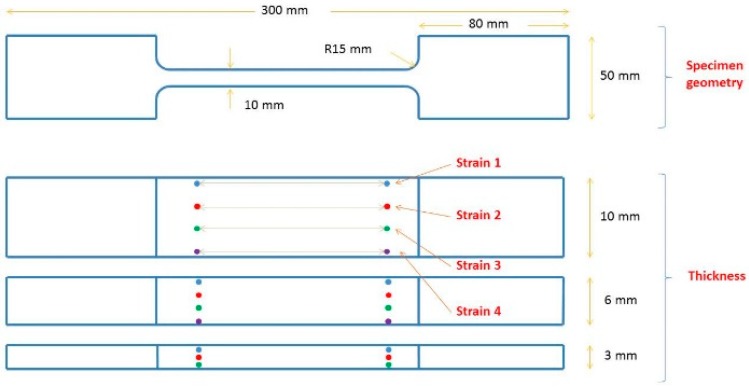
Geometry of the tensile specimens.

In this study, the tensile properties of Dyneema^®^HB26 laminate having different thickness of 3, 6, and 10 mm were investigated. The laminates were manufactured by DSM via the hot-pressing technique, which curing profile is proprietary. Dog-bone specimens (Figure 4) were waterjet cut from the as-supplied laminates. Quasi-static tensile tests were performed at room temperature using an Instron 5985 universal testing machine equipped with a 250 kN load cell, having an accuracy of ±0.5% of the displayed force. Specimens were clamped using hydraulic grips equipped with flat serrated jaw faces operated with a maximum pressure of 180 bar. Tests were performed at different cross-head displacement speeds of 1, 5 and 10 mm/min. The strain was measured using a video extensometer (Imetrum Video Extensometer) by tracking the relative displacement of three or four points marked along the thickness of the specimens within the gauge length, as shown in Figure 4.

## 3. Experimental Results

**Figure 5:**
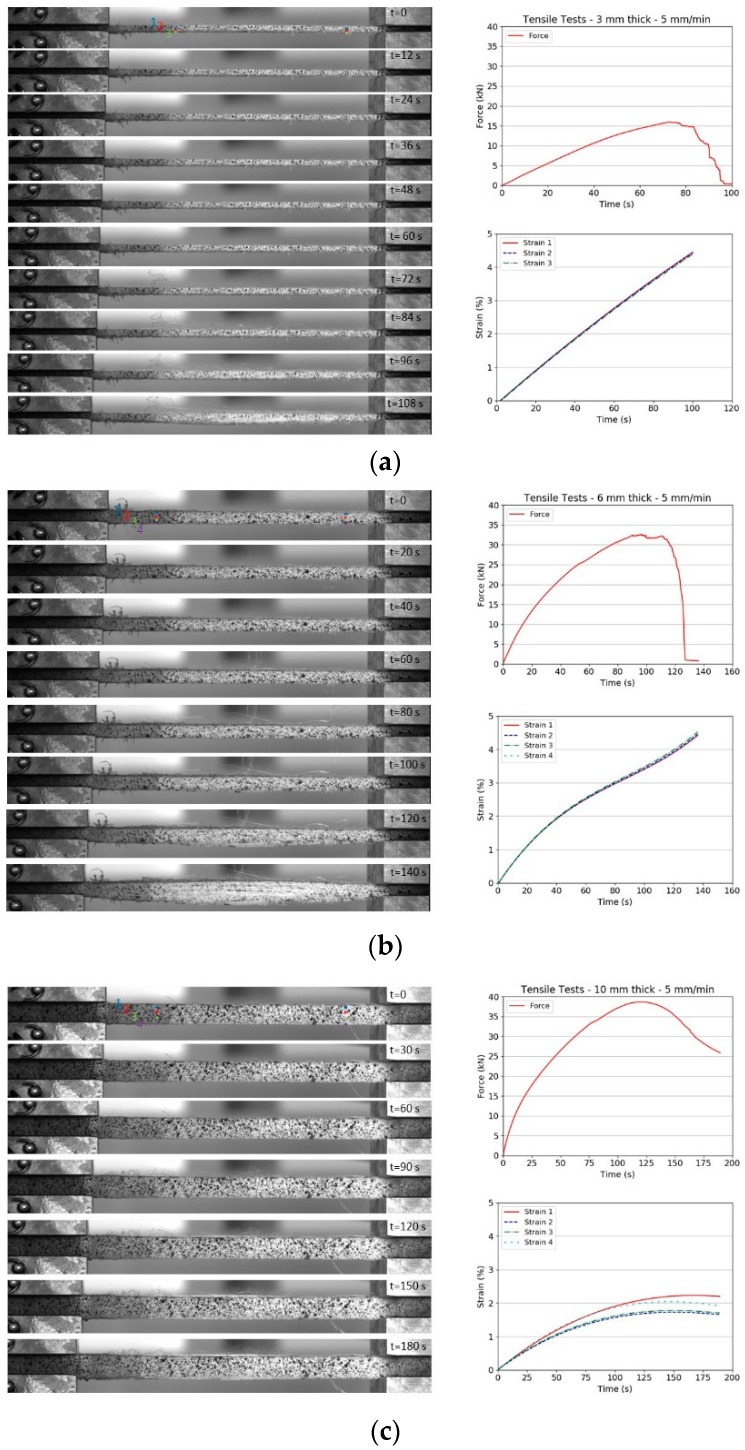
Snapshots from tensile tests and force and time history plots for Dyneema^®^HB26 laminates with different thickness: (**a**) 10 mm; (**b**) 6 mm; (**c**) 3 mm.

**Figure 6:**
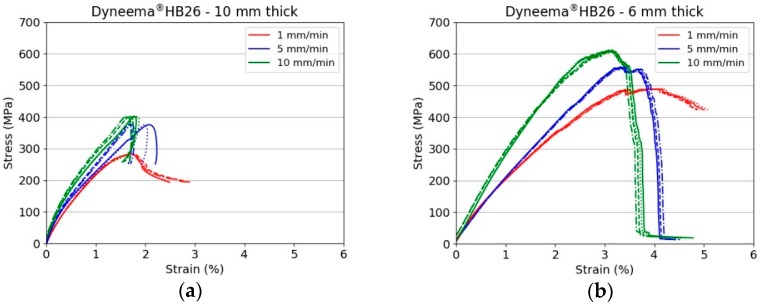

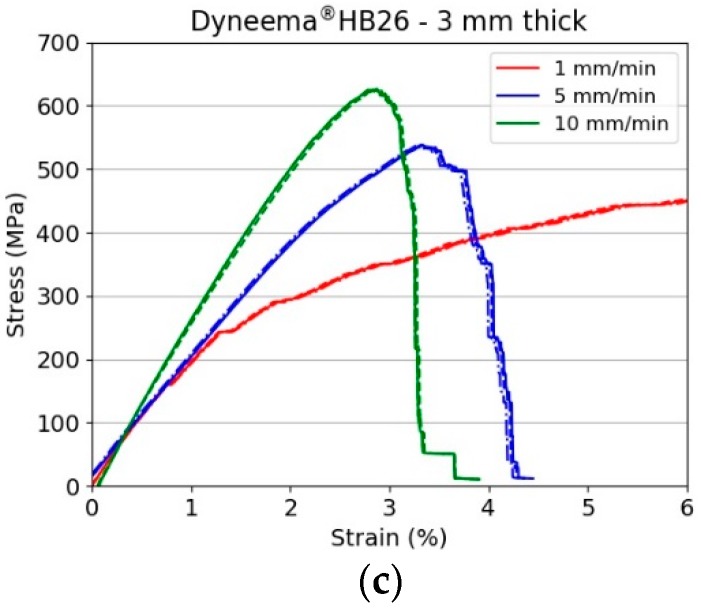
Stress vs. strain curves for Dyneema^®^HB26 laminate with different thickness: (**a**) 10 mm; (**b**) 6 mm; (**c**) 3 mm.

Figure 5 presents a series of snapshots taken during the tensile tests on Dyneema^®^HB26 laminates having different thickness (only representative 5 mm/min tests are shown). Beside the snapshots, the force vs. time and strain vs. time plots are also shown for each test. Figure 6 shows the stress vs. strain curves for Dyneema^®^HB26 laminates tested at different cross-head speeds. 

**Figure 7:**
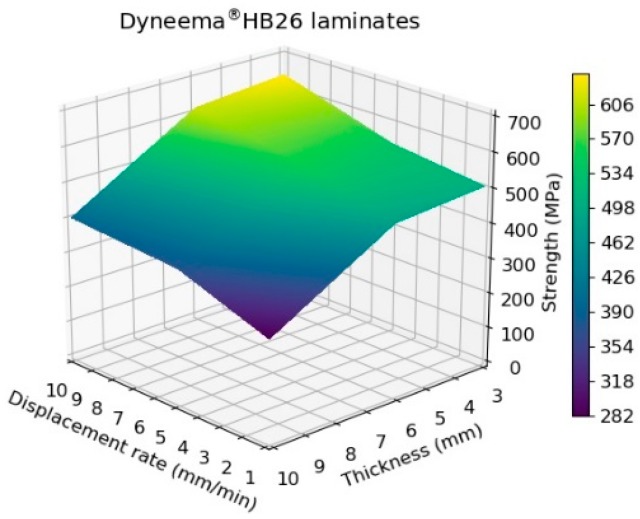
Tensile strength of Dyneema^®^HB26 laminates as function of thickness and displacement rate.

**Figure 8:**
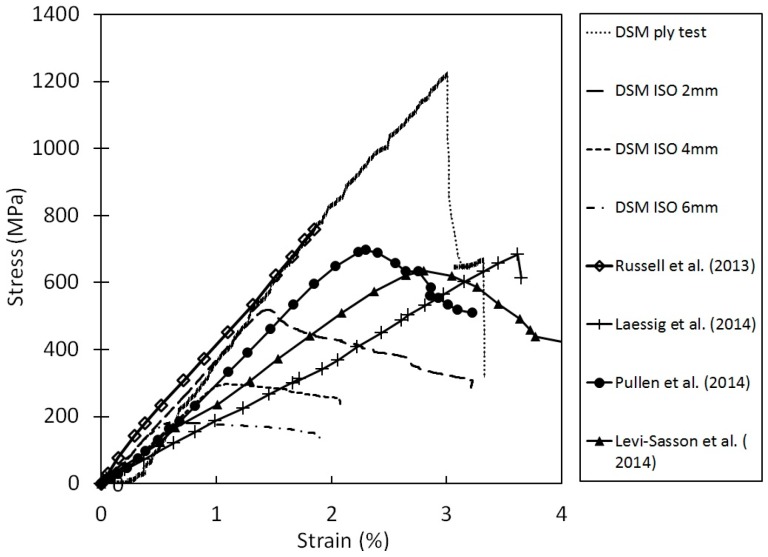
Tensile testing of Dyneema^®^ at varying thicknesses by different researchers [19].

The experimental results indicate that, for a fixed thickness, the tensile strength of the laminate increases with increasing the testing speed. While, for a constant test rate, the strength decreases with increasing the specimen thickness. However, evaluating the results plotted in Figure 7, it is possible to note that the maximum tensile strength for the 10 mm specimens ranged between 282 and 401 MPa amongst the investigated testing speeds. These values are well below the theoretical strength of the material in a laminate form (1411 MPa, considering the tensile strength of the fibres to be 3400 MPa and the laminate fibre volume fraction of 83%) and below the values reported in previous published works (Figure 2a,b). The tensile strengths for 6- and 3 mm thick specimens were as high as 539 and 638 MPa, respectively, when tested at the highest investigated displacement rate. Nevertheless, even for the thinnest specimen tested at 10 mm/min, it was not possible to match the values reported in the literature due to the difference in the specimen geometry and specimen preparation. The results are compared with a detailed review performed by DSM in Section 4. The strong dependency on the thickness is shown in Figure 8 [19].

**Figure 9:**
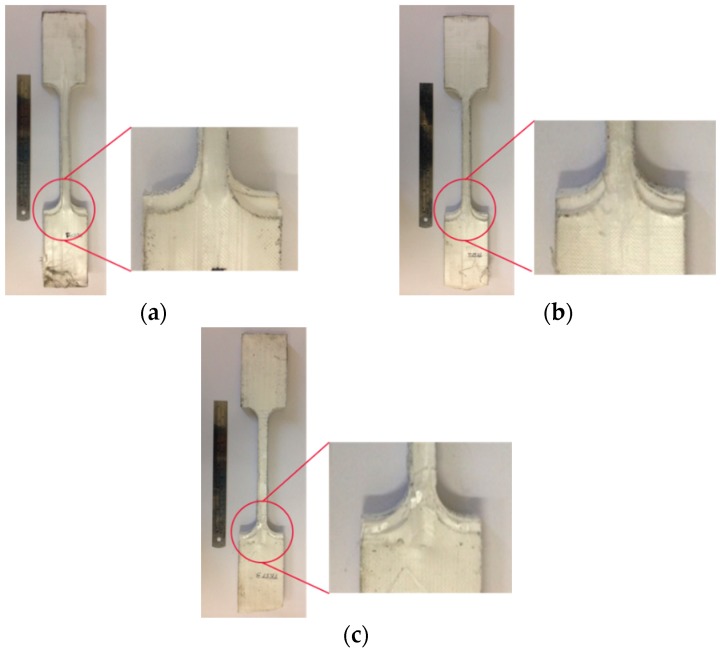
Dyneema^®^HB26 (10 mm thick) tested at different crosshead displacement rates: (**a**) 10 mm/min; (**b**) 5 mm/min; (**c**) 1 mm/min.

It can be clearly seen in Figure 9 that a separation occurred on the outermost layer from the core layers of the laminate in contact with the grips. Due to this phenomenon, the load could not be transferred from the outermost to the innermost layers by shear thus the gauge length region of the specimen could not achieve a uniform stress field at failure. At the end of the test, the edge of the outer layer of the laminate separated as much as 6 mm with respect to the edge of the specimen. The 6- and 3 mm thick specimens experienced the same layer separation, but to a lesser extent, the separation was at most 3 and 1 mm, respectively.

Examination of the strain vs. time history plot in Figure 5a indicates that the strain of the outermost points drawn along the gauge length of the specimen (Point 1 and Point 4) experienced a significantly higher strain with respect to the strain noted for the inner ones (Point 2 and Point 3). The difference in strain between the outer and inner points decreased with decreasing the specimen thickness, with the mid- and thin specimens experiencing a fairly uniform strain field through the thickness.

It is important to highlight the fact that the strain at the ultimate tensile strength $\sigma_{uts}$ for the 6 mm thick specimens was greater with respect to the strain at $\sigma_{uts}$ noted for the 3 mm specimens tested at the medium and high displacement rates, respectively. Moreover, after $\sigma_{uts}$, the fall in stress was smoother for the 6 mm specimens and steeper for the 3 mm specimens. This observation indicates that, although the specimens failed in the same macroscopic fashion, a higher extent of slippage occurred in the 6 mm thick specimens. At the slowest testing speed, it was not possible to fail specimens, which always slipped through the gripped outermost layers. The weak interfaces prevent the full transfer of load to each prepreg layer, then into the filaments, then into the macro fibril. The very low compressive stress results in a low load bearing stress, hence the use of bolts cannot eliminate these issues.

## 4. Material Model for Fibre

The morphology of UHMWPE is highly complex, however, a number of physically based models have been recently developed to explain the microstructure and the overall molecular behaviour. In this work, the continuous crystalline model is used in the development of a new continuum-based fibre material model. The fibre is assumed to be constructed from ~200 macro fibrils of different effective diameters, typically of the order of 0.1 micrometres. These macro fibrils are treated as crystalline along their entire length, with defects and amorphous regions within this structure [20]. The exact ratios of the regions are a function of the draw ratio. For a typical draw ratio of 100, the crystalline regions have a length of ~70 nm separated by disordered regions with a length of ~4 nm [4].

In the current macro fibril model, a linear stress-strain relationship to failure is assumed with the dissipated energy, area under the stress-strain curve, assumed to be only dissipated as heat in the amorphous region. When the temperature reaches the softening temperature of the Dyneema^®^, or more precisely the softening temperature of the amorphous regions, failure is deemed to have occurred via a flow process as the engineering properties are reduced to a melt or flow state. A linear relationship up to the onset of failure is assumed, however, a simple viscoelastic behaviour up to this point could also be included within the constitutive model. During a high rate event it is argued that the viscoelastic part would degenerate to linear response. This has been experimentally observed by Russell et al. [9].

### 4.1. Damage Definitions

The starting point in the development of a material model is to develop a representative volume and understand the damage mechanisms which occur within this volume. As the material model is based on a volume, it is sometimes referred to as a damage mechanics approach, since the processes are defined within a representative volume. Ultimately the volume must be linked to a finite element volume for use in the Finite Element code ls-dyna [21].

The definition of effective stress is usually derived from the principles of strain equivalence [22]:

(1) $$\bar{\sigma }=\frac{\sigma }{\left(1-d\right)},$$

where d=0 represents a virgin intact material, and d=1 the fully damaged material. The instantaneous modulus of elasticity E can be related to the undamaged modulus of elasticity $E^{0}$ using the following relationship:

(2) $$E=\left(1-d\right)E^{0},$$

The proposed laminated prepreg damage model uses this basic concept and has two damage variables per prepreg. Each prepreg within the laminate is modelled with an integration point. Tensile failure in the local 0 (or warp) and local 90 (or weft) directions is modelled with a single damage variable in each local ply direction. Namely:

- $d_{1}$ associated with the degradation of $E_{11}$ due to tensile stresses, e.g., 0 prepreg direction
- $d_{2}$ associated with the degradation of $E_{22}$ due to tensile stresses, e.g., 90 prepreg direction

**Figure 10:**
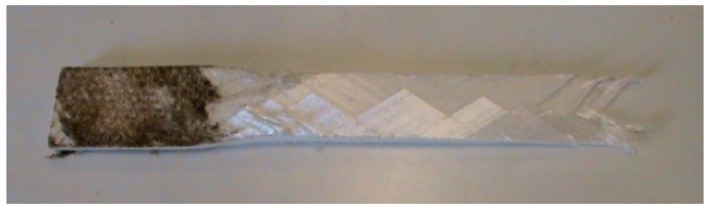
Failure of tension shear specimen.

**Figure 11:**
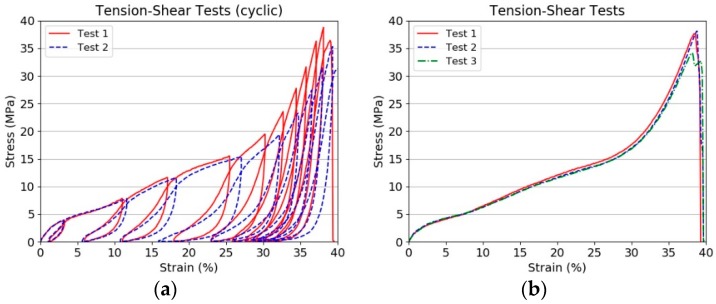
Tensile stress-strain relationship for tension shear specimen. (**a**) Cyclic tests; (**b**) Monotonic tests.

When damage is equal to 1 complete failure of the warp or weft layers has occurred at the designated laminated prepreg level, i.e., individual prepreg layers can be modelled. The G_12_ in-plane shear response is non-linear until failure and follows the measured +45/−45 tensile shear tests. A typically failed specimen is shown in Figure 10, while the cyclic tension-shear and monotonic tests are shown in Figure 11.

The shear tests clearly indicate the very weak interface and thus provides the reason for treating the in-plane and out-of-plane behaviour separately, as they are only coupled via the interface and its behaviour. In the following modelling strategy, the out-of-plane shear response *G*_23_ and *G*_13_ are assumed to be linear. Compression failure in the local directions are modelled in an elastic-perfectly plastic manner. This represents the fibre folding and kinking due to compressive loadings, typically experienced from elastic unloading waves after tensile failure has occurred along the fibre [22]. Thus, the general case for the degradation of the moduli can be defined as:

(3) $$E_{11}=\left(1-d_{1}\right)E_{11}^{0},$$

(4) $$\;E_{22}=\left(1-d_{2}\right)E_{22}^{0}$$

The through thickness behaviours are assumed to be purely elastic. Similarly the shear responses are assumed linear elastic, although the in-plane shear behaviour can be assumed to be non-linear. The response is dominated by failure of the fibre; hence, the response is approximated as linear in the investigation of the specimens.

### 4.2. Stress-Strain-Damage Relationship

The stress-strain-damage relationship follows directly from the definition of the stiffness matrix for an orthotropic material. The relationship defined by Equation (5) below must be maintained in both the undamaged and the damaged state. In addition, to prevent spurious energy generation the material stiffness matrix must be positive-definite. This leads to the inequality below, as expressed by Equation (9). The Poisson′s ratios must be degraded in a similar manner to the Young′s modulus to maintain the positive-definiteness of the material stress-strain law. For full three-dimensional (3D) response, the stress-strain relationship at a local prepreg level is defined as:

(5) σ=Cε,

where

(6) $$C=\frac{1}{N}\left[\begin{array}{cccccc} \left(1-d_{1}\right)E_{11}^{0}\left(1-\nu_{23}^{0}\nu_{32}^{0}\right) & \left(1-d_{1}\right)E_{11}^{0}\left(\left(1-d_{2}\right)\nu_{21}^{0}+\nu_{23}^{0}\nu_{32}^{0}\right) & \left(1-d_{1}\right)E_{11}^{0}(\left(1-d_{2}\right)\nu_{21}^{0}\nu_{32}^{0}+\nu_{31}^{0}) & 0 & 0 & 0\\ \left(1-d_{1}\right)E_{11}^{0}\left(\left(1-d_{2}\right)\nu_{21}^{0}+\nu_{23}^{0}\nu_{32}^{0}\right) & \left(1-d_{2}\right)E_{22}^{0}\left(1-\nu_{31}^{0}\nu_{13}^{0}\right) & \left(1-d_{2}\right)E_{22}^{0}\left(\left(1-d_{1}\right)\nu_{12}^{0}\nu_{31}^{0}+\nu_{32}^{0}\right) & 0 & 0 & 0\\ \left(1-d_{1}\right)E_{11}^{0}\left(\left(1-d_{2}\right)\nu_{21}^{0}\nu_{32}^{0}+\nu_{31}^{0}\right) & \left(1-d_{2}\right)E_{22}^{0}\left(\left(1-d_{1}\right)\nu_{12}^{0}\nu_{31}^{0}+\nu_{32}^{0}\right) & E_{33}^{0}\left(1-\nu_{12}^{0}\nu_{21}^{0}\left(1-d_{1}\right)\left(1-d_{2}\right)\right) & 0 & 0 & 0\\ 0 & 0 & 0 & NG_{12}^{*} & 0 & 0\\ 0 & 0 & 0 & 0 & NG_{23}^{*} & 0\\ 0 & 0 & 0 & 0 & 0 & NG_{31}^{*} \end{array}\right],$$

(7) $$\nu_{12}=\nu_{12}^{0}\left(1-d_{1}\right),$$

(8) $$\nu_{21}=\nu_{21}^{0}\left(1-d_{2}\right),$$

(9) $$\frac{\nu_{12}}{E_{11}}=\frac{\nu_{21}}{E_{22}},\frac{\nu_{23}}{E_{22}}=\frac{\nu_{32}}{E_{33}},\frac{\nu_{31}}{E_{11}}=\frac{\nu_{13}}{E_{33}},$$

(10) $$N=1-\nu_{12}\nu_{21}-\nu_{23}\nu_{32}-\nu_{31}\nu_{13}-2\nu_{21}\nu_{32}\nu_{13}>0\;\sigma =\left[\begin{array}{c} \sigma_{11}\\ \sigma_{22}\\ \sigma_{33}\\ \sigma_{12}\\ \sigma_{23}\\ \sigma_{31} \end{array}\right],\;\varepsilon =\left[\begin{array}{c} \varepsilon_{11}\\ \varepsilon_{22}\\ \varepsilon_{33}\\ 2\varepsilon_{12}\\ 2\varepsilon_{23}\\ 2\varepsilon_{31} \end{array}\right]$$

The modelling approach can be implemented into a solid element formulation within the ls-dyna finite element code. The through thickness properties are assumed linear, as are the out-of-plane shear properties. During unloading from a stationary condition, the damage does not increase, unless the stress remains above the damage stress threshold. As damage occurs within each prepreg layer, the response is plane stress within the individual layers. The interface is assumed to be weak and will readily fail. Hence an appropriate failure relationship must be introduced into the model to account for such a response. The use of contact logic between layers is described in the coupon modelling section.

### 4.3. General Plane Stress Stress-Strain-Damage Relationship

The general plane stress stress-strain relationship for the damage model can be derived directly from Equation (5). This is shown in Equation (11):

(11) $$\dot{\sigma }=C\dot{\varepsilon }+\beta \dot{C}\varepsilon ,$$

Equation (11) can be expanded into incremental form to include a permanent or damage strain component. The magnitude of the permanent damage strain can be determined via material constants β. Cross-coupling and interaction terms are not considered in the present formulation. The stress-strain-damage relationship is hence defined by Equation (12):

(12) $$\dot{\sigma }=C\dot{\varepsilon }+{\dot{\sigma }}_{ir},$$

with

(13) $${\dot{\sigma }}_{ir}=\left(\begin{array}{c} -\beta_{1}\sigma_{11}\frac{d_{1}}{\left(1-d_{1}\right)}\\ -\beta_{2}\sigma_{22}\frac{d_{2}}{\left(1-d_{2}\right)}\\ 0 \end{array}\right),$$

**Figure 12:**
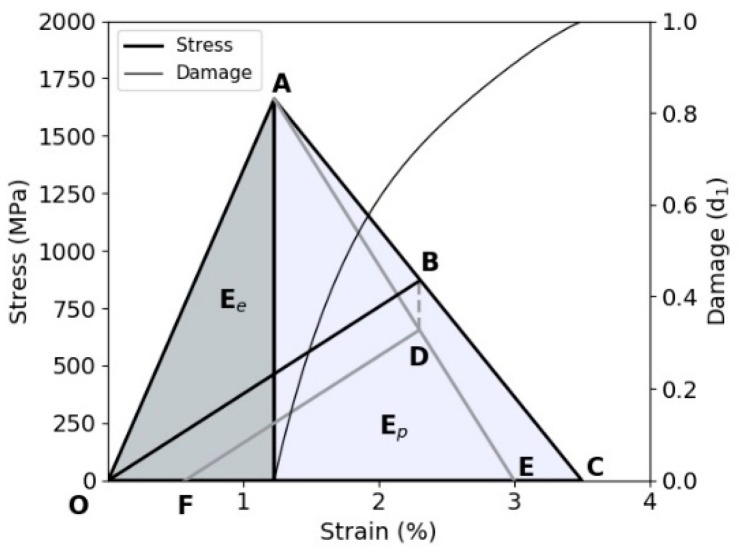
Constitutive model behaviour in tensile failure modes [22] (permanent strain (OF); irreversible stress (BD)).

The $\beta_{i}$ terms in the above equation control the amount of residual permanent strain (plastic strain). Consider the unloading point B in Figure 12; with $\beta_{i}=1$ the unloading path leads directly to the origin with no residual plastic strain, while a value of $\beta_{i}>1$ results in a positive residual plastic strain, i.e., path BDF, as the strain-softening line AC has now moved to position AE to accommodate the additional stress reduction. A value of $\beta_{i}<1$ is not permitted, as this would indicate an unrealistic negative permanent strain. In the present formulation for the irreversible stress, ${\dot{\sigma }}_{ir}$, second order terms are neglected.

### 4.4. Work Dissipated

The implemented work dissipation is also calculated based on the damage and damage rate. The work dissipation is not directly used within the stress update procedure. The work dissipation ${\dot{W}}_{i}$ for a damage rate ${\dot{d}}_{i}$ is given by Equation (14) [22]:

(14) $${\dot{W}}_{i}^{n}=\frac{\left(2\beta_{i}-1\right)}{2}\frac{\sigma_{ii}^{2}}{E_{ii}^{0}{\left(1-d_{i}\right)}^{2}}{\dot{d}}_{i}^{n},$$

where *n* denotes the *n*th time step or load increment. Clearly the total energy dissipated can be predicted for a specific volume of material.

### 4.5. Permanent Plastic Strain

The total strain is the sum of permanent (plastic) and elastic strain. From the stress-strain curve, it can be shown that the plastic strain (OF), Figure 12, is given by Equation (15):

(15) $${\dot{\varepsilon }}_{pl,i}=\left(\beta_{i}-1\right)\frac{\sigma_{ii}}{E_{ii}^{0}{\left(1-d_{i}\right)}^{2}}{\dot{d}}_{i},$$

The cumulative permanent strain is trivially defined by Equation (16):

(16) $$\varepsilon_{pl,i}^{n+1}=\varepsilon_{pl,i}^{n}+\Delta \varepsilon_{pl,i}^{n+1},$$

where *n* represents the *n*th timestep or load increment. Figure 12 illustrates the bilinear constitutive model where AC relates to $\beta_{i}=1.0$ and AE, when $\beta_{i}>1$. The greater the value of $\beta_{i}$, the greater the magnitude of the irreversible stress BD, and hence the permanent strain OF. The “plastic strain” that is defined in this paper results from residual deformation formed during damage evolution. It is clear that the $\beta_{i}$ constants can be derived from experimental fibre cyclic permanent strain versus damage plots.

### 4.6. Thermal Softening

The final equation necessary to complete the description of the fibril and fibre is the relationship for the temperature change during the deformation up to failure. No coupled thermo-mechanical finite element code is used, hence a simple adiabatic temperature change is assumed, which follows the assumption that all the irreversible work from Equation (17) is dissipated as heat:

(17) $${\dot{W}}_{i}^{n}=v_{f}\rho C_{v}{\dot{T}}_{i}^{n},$$

where 𝜌 is the density of the material and $C_{v}$ the specific heat. The volume fraction $v_{f}$ defines the amorphous material in the same unit volume as the irreversible damage. Based on existing nanoscale models [4], the total length of the representative volume would have crystalline regions with a length of ~70 nm separated by disordered regions with a length of ~4 nm thus a total length of ~74 nm. Hence, as a volume fraction the representative volume of amorphous material is defined as simply (4/74). The volume fraction $v_{f}$ is thus defined as (4/74) × (0.50) × 0.83 and accounts for the volume of amorphous material, which is heated and based on 50% of the prepreg layer, which is loaded in tension and has a volume fraction of fibre in the loaded layer of 83%. The current temperature during the damage process can be trivially calculated from Equation (17) following an incremental approach, expressed by Equation (18):

(18) $$T_{i}^{n+1}=T_{i}^{n}+\Delta T_{i}^{n+1},$$

By equating the external work, Equation (14), to the internal work, Equation (17), the adiabatic system is completed defined. The approaches are defined in such a manner that the damage evolution reaches 1 when the temperature also reaches the softening temperature. At this point, the integration point, and hence element, is removed within the Finite Element analysis indicating tensile failure has occurred. This is required to prevent excessive drawing of the finite element which has failed.

### 4.7. Damage Evolution for Tensile Direct Stresses

Failure in both the 0- and 90-degree directions is formulated in a similar manner. No cross coupling between the 0- and 90-degree plies at failure is included. A linear behaviour until failure is assumed for the macro fibril based on available evidence [23]. Once the initiation (failure) stress is reached, damage initiates and stress is gradually reset to zero in either the 0- or 90-degree directions as the damage reaches a value of one and the temperature reaches the softening temperature. Therefore, element deletion represents a physical failure in the laminate, if damage reaches 1 in either the 0- or 90-degree directions for all integration points within an element (i.e., all laminae layers have failed if a multiply integration points are used within a single element). For the 0- and 90-degree fibre failure case (i=1, 2), the damage evolution equation is defined as OAE in Figure 12, when no permanent strain is present:

(19) $$d_{i}=\frac{\varepsilon_{\max,i}}{\left(\varepsilon_{\max,i}-\varepsilon_{0,i}\right)}\left[1-\frac{\varepsilon_{0,i}}{\varepsilon_{ii}}\right],$$

where $\varepsilon_{\max,i}$ is the strain at zero stress and damage = 1, and $\varepsilon_{0}$ is the strain at maximum stress (failure stress) and damage = 0. The only parameters required for this evolution model are these two strain constants, which define the total energy dissipated, i.e., the area under the stress-strain curve. Equation (20) can be converted into an incremental form, which has been implemented into the ls-dyna code. Such an approach is commonly used for carbon composites [22] and the compact tension test procedure used to determine the area under the stress-strain curve, due to fibre pull-out and fracture [24].

(20) $$\Delta d_{i}=\frac{\varepsilon_{\max,i}}{\varepsilon_{\max,i}-\varepsilon_{0,i}}\left[\frac{\varepsilon_{0,i}}{\varepsilon_{ii}^{2}}\right]\Delta \varepsilon_{ii},$$

The constants $\varepsilon_{max,i}$ and $\varepsilon_{0,i}$ must be chosen such that the temperature has reached the softening temperature at the same time as the structural analysis calculation has completed the structural softening process with a damage of 1. In the current formulation, the volume is the finite element volume. The damage evolution can then be trivially stated as:

(21) $$d_{i}^{n+1}=d_{i}^{n}+\Delta d_{i}^{n+1},$$

where *n* represents the *n*th time step or load increment.

In the current formulation, the failure displacement of the finite element i.e., $\varepsilon_{\max,i}$ is adjusted at the element level so that the energy dissipated for the volume of the finite element will cause the temperature to reach the softening temperature, independent of element size. Thus, the irreversible damage energy is linked directly to the amorphous regions in the same volume. It may be necessary to include a characteristic “length” or volume of material in the process zone such that softening only occurs within a characteristic zone independent of the finite element. At the moment the logic is that the elements are sufficiently small that the adiabatic assumption is valid, a common assumption in the modelling of thermal softening due to plastic work [21]. Experimentally the volume of material heated within the same volume as the finite element may not increase to the softening temperature in a uniform manner as the distribution of amorphous material will not necessarily be uniform. If a “hot spot” develops it will tend to localise in this amorphous macro fibril region and failure occurs. This can be defined as a characteristic “length” within the constitutive framework. However, the relationships between the spacing of the amorphous regions and the relationship between the softening and melting temperatures for the macro fibril require a detailed molecular modelling approach, especially as the chain pull-out process needs to be explicitly modelled. However, once softening occurs, the shear strength of the material will reduce dramatically and the fibre will fail in a drawing process, as observed in Figure 3a. If a material characteristic “length” or volume were introduced, it would be trivial to include within a mesh independent solution by maintaining constant energy dissipation independent of volume.

**Figure 13:**
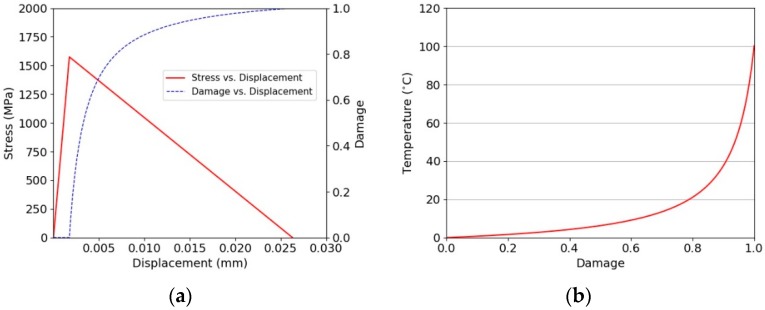
Constitutive model. (**a**) Behaviour for single element test; (**b**) temperature vs. damage.

**Figure 14:**
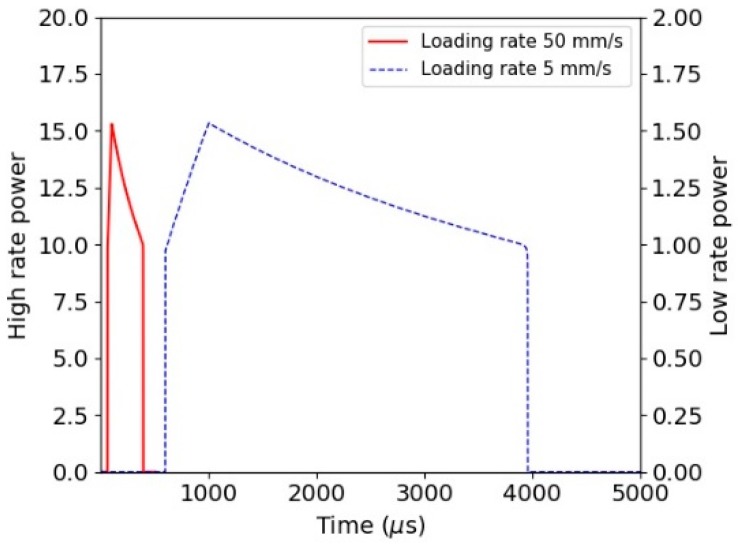
Power dissipated for single element tests (units of power are per unit volume).

The implementation within the ls-dyna explicit code is demonstrated using a simple element cube of length 50 µm under a uniform displacement loading. The corresponding stress-displacement relationship for a single ply direction is shown in Figure 13a, with the corresponding damage-temperature plot shown in Figure 13b. The rate of damage growth is a function of the strain, as shown by Equation (20). The power dissipated for the simple cube is plotted in Figure 14. It can be clearly seen that the energy dissipated as a function of the rate of loading is not capped in this figure. The rate of molecular pull-out from the chain within the amorphous region of the macro fibril may potentially be limited. This can be investigated using molecular modelling techniques. Clearly, including such a limit on the rate of chain pull-out will be equivalent to including a rate sensitivity into the constitutive model.

## 5. Specimen Modelling

**Figure 15:**
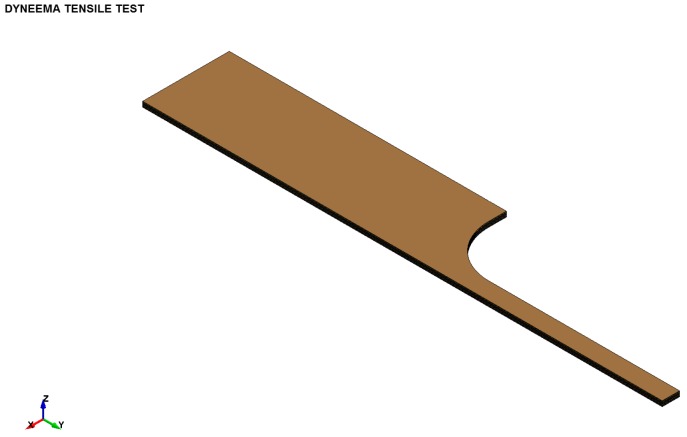
Finite element model (eighth symmetry) with ten layers.

The specimens were modelled with single integration solid finite elements in eighth symmetry. This model is composed of equivalent prepreg layers of 0.5 mm thick sub-laminates bonded together using the surface-to-surface contact login in ls-dyna, which includes initiation strengths (30 MPa), equivalent energy dissipated to propagate (2 kJ/m^2^), and static/dynamic frictional coefficients within the interface contact logic. The frictional part of the surface-to-surface contact activates for post-failure sliding. A pre-stress is applied to the tabbed region to represent the preload applied during the test. Each sub-laminate with the specimen is modelled with the constitutive material model outlined in Section 4. Figure 15 illustrates the generic model for the 10 mm specimen. The dynamic friction coefficient between the layers is altered to understand the importance and how the specimen stress-strain derived from the gauge length changes. Each finite element is modelled with a linear stress-strain law up to damage initiation based on the macro fibril constitutive model outlined in Section 4. No visco-elastic behaviour is included for the pre-failure behaviour.

**Figure 16:**
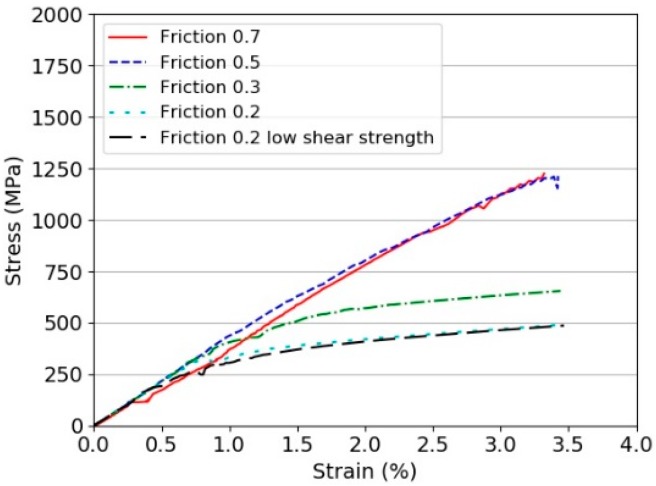
Virtual tensile testing of a Dyneema^®^HB26 laminate specimen (thickness 10 mm) with different static and dynamic frictional coefficients.

The results for different interface strengths and friction coefficients are shown in Figure 16. This can be compared with the experimental results observed from other researchers, including the current tests, as presented in Figure 8.

The comparison clearly shows the same trend and that the laminate stress-strain curve does not reflect the real strength of the fibres due to the very poor interfaces, both in-plane and out-of-plane. The initiation strength was reduced to 10 MPa to understand the sensitivity of this strength, which appeared to have only a small effect on the resulting predicted stress-strain curve of the specimen.

**Figure 17:**
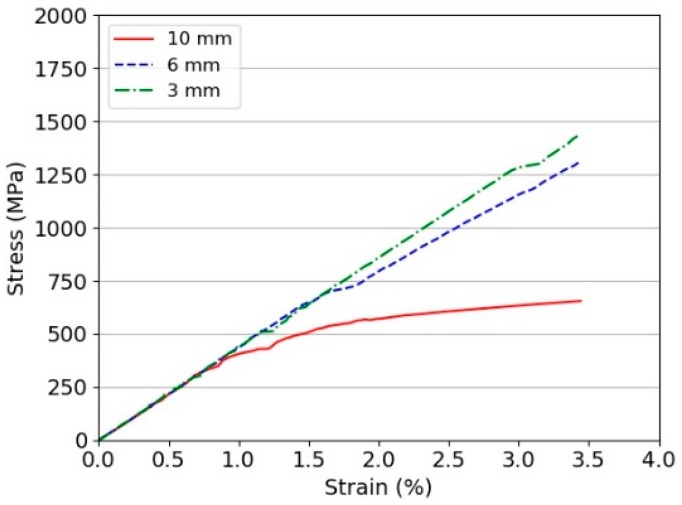
Virtual tensile testing of a Dyneema^®^ laminate specimen (thickness 3, 6, and 10 mm) with the same static and dynamic frictional coefficients (0.3).

A similar analysis was performed with a constant interface behaviour, but matching the three thicknesses tested. Specifically 10, 6, and 3 mm. The behaviour is shown in Figure 17. Clearly the same effect is observed in which the reduced thickness generates the highest strength. As the interfaces are removed and the scale reduced the ultimate strength will converge to the fibril strength.

**Figure 18:**
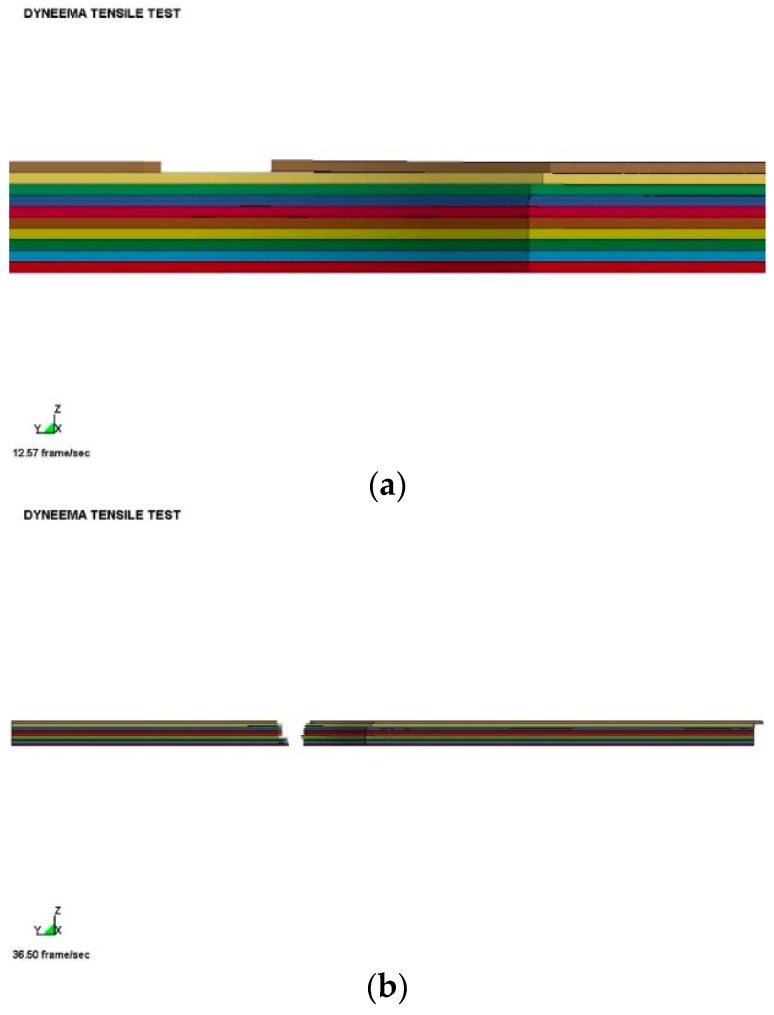

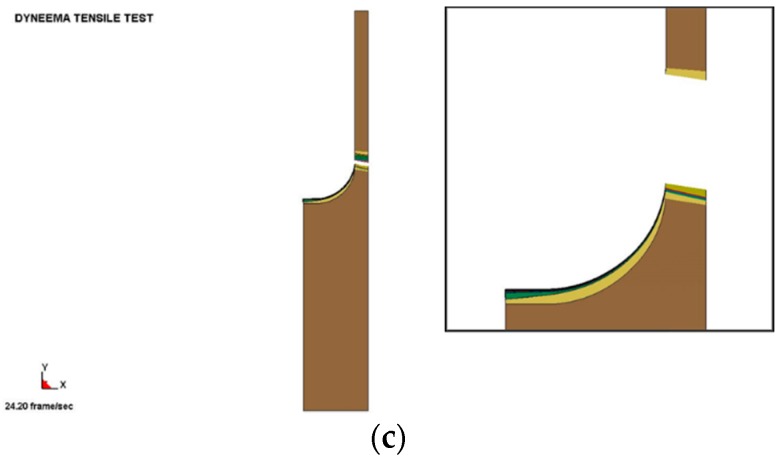
Finite Element model with ten layers. (**a**) Partial failure; (**b**) Complete failure; (**c**) Complete failure and slippage (insert showing close-up).

The sensitivity parameter within the contact interface was the dynamic and static friction coefficients, which would indicate the ability to continue to transfer the load once a layer unloaded, due to failure, was very important. Ideally the frictional coefficients used with the interface could be made a function of rate, temperature, both ambient and interface generated, and confining pressure. This is not available in ls-dyna, but could be implemented via a user-defined interface. It is believed that the interfaces are critical in transferring the load between layers. It also is believed that such an approach can be used to optimise the interfaces for ballistic impacts. Figure 18a shows a typical failure when the core plies cannot be failed, while Figure 18b,c present the deformation for complete failure. It can be clearly seen that all plies are not uniformly loaded and the progressive nature of the failure results in the lower strengths compared with the expected strengths based on simple theory of mixtures.

Hence the use of strengths derived from laminate tests in a numerical modelling strategy would underestimate the real strength of a Dyneema^®^ laminate. It is proposed that a more effective strength should be based on macro fibril strength and scaled up using the theory of mixtures, which is believed to be the more realistic strength which an impactor may experience during an impact event.

## 6. Conclusions

In this paper, we address the difficulty in inducing tensile failure in Dyneema^®^ laminates. We experimentally and computationally demonstrate that load from the outer layers is not able to reach the core layers. This phenomenon is exacerbated in thick specimens tested at low strain rates. The experimental programme also highlighted a viscoelastic effect at pseudo static test rates, which is believed to be associated with the interface and the load transfer to the core plies.

A new constitutive material model for the macro fibril has been developed based on the softening characteristic of the amorphous regions within the fibre. The fibre modelling approach is combined with an interface modelling approach to understand the important characteristics of the Dyneema^®^ interfaces. Ultimately such an approach can be used in inverse fashion to determine the optimum interface to maximise the engagement of fibres within the laminate but still allowing the fibres to work in a tensile mode. The current model lacks of a coupled rate, pressure, temperature and friction contact logic, which do not allow heat generation at the interfaces, and is limited to the prediction of the material properties under quasi-static regimes. The development of an improved interface modelling approach which includes rate, pressure and thermal softening could lead to more effective designs of polymer armours as the interface could be tailored for the specific threat under consideration. An Equation of State can be easily added to allow the prediction of the ballistic properties for high velocity/hyper velocity impact cases.

The key conclusion highlights that the intrinsic macro fibril strength should be used in numerical studies. The fibril strength is a function of the amorphous volume within the fibril. The experimental behaviour has been simulated by modelling the interface between laminate plies and the macro fibril failure. The weak interfaces from fibril scale to the laminate scale makes the testing of fibres and laminates very difficult. Hence it is proposed that the fibril strength should be used for all modelling.
